# Origin of the High Density of Oxygen Vacancies at the Back Channel of Back-Channel-Etched a-InGaZnO Thin-Film Transistors

**DOI:** 10.3390/mi15030400

**Published:** 2024-03-16

**Authors:** Shimin Ge, Juncheng Xiao, Shan Li, Dong Yuan, Yuhua Dong, Shengdong Zhang

**Affiliations:** 1 School of Electronic and Computer Engineering, Shenzhen Graduate School, Peking University, Shenzhen 518055, China; geshimin@pku.edu.cn (S.G.); xiaojuncheng@stu.pku.edu.cn (J.X.); 2 TCL China Star Optoelectronics Semiconductor Display Technology Co., Ltd., Shenzhen 518132, China; lishan@tcl.com (S.L.); yuandong3@tcl.com (D.Y.); dongyuhua1@tcl.com (Y.D.); 3 School of Integrated Circuits, Peking University, Beijing 100871, China

**Keywords:** a-IGZO TFTs, back-channel damage, device stability, oxygen vacancy

## Abstract

This study reveals the pronounced density of oxygen vacancies (Vo) at the back channel of back-channel-etched (BCE) a-InGaZnO (a-IGZO) thin-film transistors (TFTs) results from the sputtered deposition rather than the wet etching process of the source/drain metal, and they are distributed within approximately 25 nm of the back surface. Furthermore, the existence and distribution depth of the high density of Vo defects are verified by means of XPS spectra analyses. Then, the mechanism through which the above Vo defects lead to the instability of BCE a-IGZO TFTs is elucidated. Lastly, it is demonstrated that the device instability under high-humidity conditions and negative bias temperature illumination stress can be effectively alleviated by etching and thus removing the surface layer of the back channel, which contains the high density of Vo defects. In addition, this etch method does not cause a significant deterioration in the uniformity of electrical characteristics and is quite convenient to implement in practical fabrication processes. Thus, a novel and effective solution to the device instability of BCE a-IGZO TFTs is provided.

## 1. Introduction

Amorphous oxide thin-film transistors (TFTs), represented by amorphous indium-gallium-zinc-oxide (a-IGZO) TFTs, have attracted much attention for applications in large-area electronics such as active matrix displays due to their high mobility, low off-current, and low-cost production process [1,2,3,4]. Among the various device structures, the back-channel-etched (BCE) structure is widely used in a-IGZO TFTs due to its lower mask number and simple production process [5]. However, the electrical instability of BCE a-IGZO TFTs is still a crucial challenge [6,7,8,9]. It has been reported that the BCE a-IGZO TFTs in particular suffer serious degradation under high temperature and high humid storage (HTHHS) conditions [10,11,12], positive bias temperature stress (PBTS) [13], and negative bias temperature illumination stress (NBTIS) [14,15,16].

It is well known that BCE a-IGZO TFTs have a pronounced density of oxygen vacancies (Vo) at the back channel, which is believed to be the major origin of their electrical instability [17,18]. Two mechanisms have been proposed to account for the formation of a high density of Vo defects at the back channel. One is that the chemical reactions between the etchant and metal–oxygen bonds of a-IGZO film could occur and induce the Vo defects during the wet etching process of source/drain (S/D) electrodes [19]. The other one is that the magnetron sputtered deposition process of S/D metal could lead to the formation of Vo defects due to the strong ion bombardment [20]. However, these two mechanisms have not yet been experimentally confirmed, and the origin of the high density of Vo defects is still unclear.

In addition, how Vo defects at the back channel lead to the instability of BCE a-IGZO TFTs has not been clarified clearly. Furthermore, BCE a-IGZO TFTs still suffer more serious stability degradation than etch-stop-layer (ESL) structured TFTs, although the passivation deposition and annealing process could repair the Vo defects at the back channel [21,22,23]. Thus, the instability of BCE a-IGZO TFTs continues to be a critical issue.

In this work, the origin of the high density of Vo defects at the back channel of BCE a-IGZO TFTs is investigated and clarified. In addition, a physical model is proposed to explain how the generated Vo defects at the back channel lead to the device instability. In addition, an effective way of alleviating the device instability of BCE a-IGZO TFTs is proposed and verified.

## 2. Materials and Methods

The experimental work in this study had two parts; the first was the investigation of electrical characteristics of a-IGZO films. a-IGZO films with a thickness of 80 nm were deposited on the glass substrate via alternating current (AC) magnetron sputtering with oxygen/argon gas flow-rate ratio of 30/70 at room temperature, and annealed at 350 °C for 1 h. The atomic ratio of In:Ga:Zn in the oxide ceramic target was 1:1:1. Then, the molybdenum/copper (Mo/Cu) stacked films with a thickness of 30/550 nm were deposited on some of the a-IGZO films by means of sputtering to simulate the deposition process of S/D metal in the fabrication of BCE a-IGZO TFTs. After that, a H_2_O_2_-based copper etchant with or without fluorine (F)was used to etch the Mo/Cu films to simulate the wet etching process of S/D metal. The etching rates of the a-IGZO films etched using the copper etchant with and without fluorine were measured to be about 0.03 and 0.4 nm/s, respectively. Some of the other annealed a-IGZO films without Mo/Cu deposition step were etched using the wet etching process in order to study the effect of only the wet etching process. Finally, the sheet resistance (R_s_) values of the a-IGZO films treated via the above different processes were measured using a four-probes resistance tester. Three samples for each case were prepared with the error of film thickness of less than 4 nm (±2 nm).

**Figure 1:**
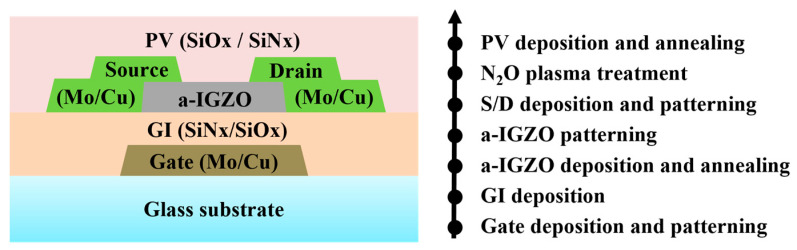
The schematic cross-section and the main process flow of the fabricated BCE structured a-IGZO TFTs in this work.

The second part of the experiment was the fabrication of BCE a-IGZO TFTs. The schematic cross-section of the fabricated TFTs in this work is shown in Figure 1. The fabrication process was as follows: the Mo/Cu stacked films with thicknesses of 30/550 nm were firstly deposited and patterned to form the gate electrodes. The SiN_x_/SiO_x_ dual-layer with a thickness of 300/100 nm was then deposited at 360 °C by means of plasma-enhanced chemical vapor deposition (PECVD) to form the gate insulator (GI). After that, the a-IGZO films with different thicknesses were deposited via sputtering and annealed under the same conditions as in the first part of the experiment, and then patterned through wet etching using oxalic acid. Following that, the Mo/Cu stacked films with thicknesses of 30/550 nm were deposited and patterned through wet etching using H_2_O_2_-based copper etchant to serve as the S/D electrodes. Next, 10 s of N_2_O plasma treatment was applied and then the SiO_x_/SiN_x_ dual-layer as the passivation (PV) layer with a thickness of 200/100 nm was deposited by means of PECVD at 230 °C. Finally, one more annealing process was performed at 300 °C for 1 h.

The transfer characteristics of the fabricated BCE a-IGZO TFTs were measured using a Keithley 4200S semiconductor parameter analyzer (Tektronix, Beaverton, OR, USA). The PBTS measurements were carried out under a gate voltage (V_g_) of +30 V at 60 °C, and the NBTIS measurements were performed under a V_g_ of −30 V at 60 °C with an illumination of 4500 nits (white LED light). The threshold voltage (V_th_) is defined as the V_g_ value at which the normalized drain current (I_d_) is 10^−9^ A.

## 3. Results

The measured R_s_ values of the a-IGZO films after different treatment processes are listed in Table 1. The results show that the annealed a-IGZO films have a high R_s_ value of around 10^9^ Ω/sq. For the annealed a-IGZO films etched with only the wet etching process, the R_s_ value does not show an obvious change after the wet etching process, regardless of the use of F-free or F-containing copper etchant. However, for the annealed a-IGZO films which experienced the sputtered deposition of S/D metal, the R_s_ value is reduced to about 10^4^ Ω/sq after the wet etching of S/D metal using conventional F-free copper etchant. Therefore, it can be reasonably inferred that the remarkable reduction in R_s_ is attributed to the sputtered deposition rather than the wet etching process of S/D metal. It is well known that the amounts of hydrogen and Vo defects are the two main factors affecting the carrier concentration in a-IGZO films. Hydrogen was not intentionally introduced during the formation of S/D electrodes in this work. Thus, the high conductance of the a-IGZO film-4 in Table 1 is due to the formation of a high density of Vo defects during the sputtered deposition process, in which the metal–oxygen bonds of a-IGZO films are broken due to the strong ion bombardment [24]. In addition, the H_2_O_2_-based copper etchant is unlikely to generate the pronounced Vo defects during the wet etching process of S/D electrodes.

**Figure 2:**
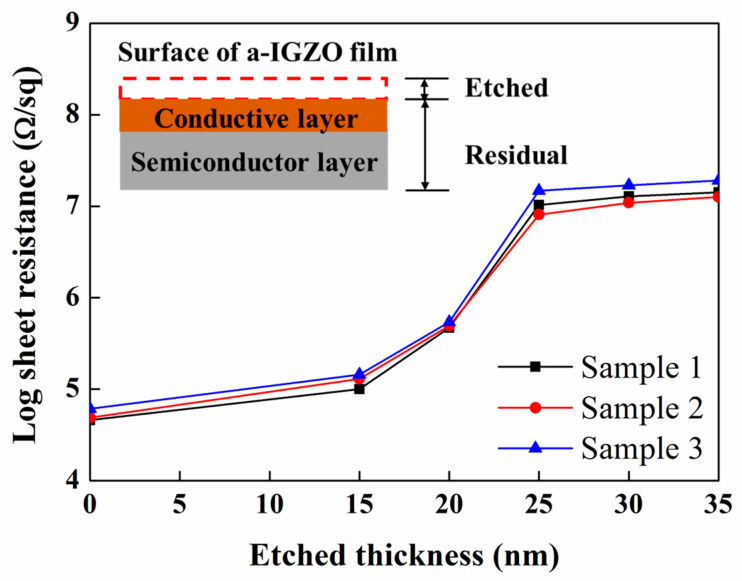
The R_s_ values of the residual a-IGZO film with various etched thicknesses. The inset shows the schematic cross-section of residual a-IGZO film after being etched in dilute hydrochloric acid. Lines are just to guide the eyes.

To clarify the depth distribution of the generated Vo defects caused by the sputtered deposition of S/D metal, we evaluated the R_s_ depth profile of a-IGZO film-4 in Table 1. The surface of a-IGZO film was repeatedly etched in dilute hydrochloric acid [25] to obtain the thin film. Figure 2 shows the R_s_ values of the residual a-IGZO film with various etched thicknesses, and the inset shows the schematic cross-section of the residual a-IGZO film after being etched in dilute hydrochloric acid. The R_s_ values of the residual a-IGZO film were measured after each etching step. The R_s_ value of the a-IGZO film-4 is initially about 10^4^ Ω/sq and increases as the etched thickness increases. When the etched thickness is over 25 nm, the R_s_ value is about 10^7^ Ω/sq and nearly remains constant with a further increase in the etched thickness. Obviously, the remarkable increase in R_s_ from 10^4^ to 10^7^ Ω/sq should be ascribed to the significant difference in conductance between the surface and bulk of the a-IGZO films, instead of the 30% decrease in film thickness. These results suggest that a high density of Vo defects are distributed within approximately 25 nm of the surface of the a-IGZO films. In addition, the high R_s_ value of around 10^7^ Ω/sq of a-IGZO film-5 shown in Table 1 should be attributed to the removal of the Vo defects in the surface layer by the F-containing etchant, which has a relatively high etching rates of 0.4 nm/s and could remove the surface layer of a-IGZO film after removing the S/D metal films by means of over-etching.

**Figure 3:**
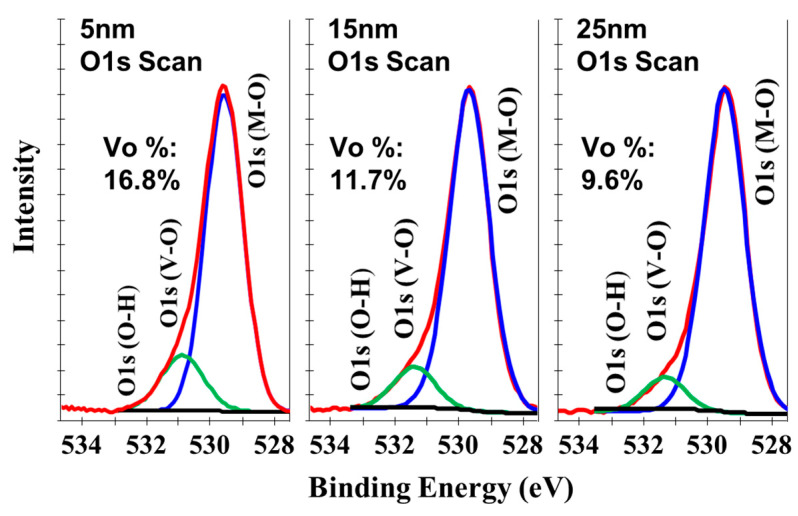
XPS spectra of the O 1s signal of a-IGZO film-4 in Table 1 at the detection depths of 5, 15 and 25 nm.

In order to further verify the existence and distribution depth of the high density of Vo defects, we performed XPS spectra analyses on the surface and bulk of a-IGZO film-4 in Table 1. Figure 3 shows the XPS spectra of the O 1s signals at the detection depths of 5, 15, and 25 nm. The broad peak of the O 1s signal is divided into two peaks centered at around 529.8 eV and around 531.6 eV, which are associated with the lattice oxygen and Vo defects in the a-IGZO films, respectively [25]. The larger area percentage of the peaks related to Vo defects at the detection depth of 5 nm suggests a significant increase in the number of Vo defects at the surface layer. In addition, the percentage of Vo defects decreases from 16.8% to 9.6% with increase in the detection depth from 5 to 25 nm. Meanwhile, the percentage of Vo defects at the detection depth of 25 nm is close to that of a-IGZO film-1 in Table 1, with a percentage of 8.4%. These results verify that a high density of Vo defects are formed at the surface of a-IGZO films after the sputtered deposition of S/D metal.

Furthermore, the measured R_s_ value of a-IGZO film-4 in Table 1 after applying 10 s of N_2_O plasma treatment was around 10^9^ Ω/sq. This result suggests that the shallow donor state of Vo defects can be repaired during the passivation process, which has also been verified by the obtained positive V_th_ for BCE a-IGZO TFTs in previous research [21,22,23]. However, the device stability of BCE a-IGZO TFTs still suffers more serious degradation under high-humidity or NBTIS conditions compared to etch-stop-layer (ESL) structured TFTs. Hence, there are a large number of remaining unrepaired deep-state Vo defects. It is noted that there are two different oxygen vacancy states: one is the shallow donor state of Vo defects with two electrons provided (Vo^2+^), the other one is deep-state Vo defects with two localized electrons (Vo^0^) [26]. It is speculated that the passivation process could repair most of the shallow-state Vo^2+^ defects but only some of the deep-state Vo^0^ defects.

However, the mechanism by which the Vo^0^ defects at the back channel deteriorate the device stability of BCE a-IGZO TFTs has not been clearly elucidated. In this work, the following model is proposed. A high density of Vo^0^ defects could encourage the hydrogen (H) from the passivation layer and the humid environment to fill Vo^0^ defects [27,28,29], forming two kinds of substitutional H states shown in the following reaction equations

Vo^0^ + 2H → Vo^2+^(2H) + 2e^−^ (1)

Vo^0^ + 2H → Vo^0^(2H) (2)

where Vo^0^(2H) and Vo^2+^(2H) represent the neutrally and positively charged substitutional H states at the Vo^0^ site, respectively, while e^−^ denotes the free electron. As shown in Equation (1), two free electrons are formed after Vo^0^ is substituted by H. Thus, the increasing concentration of free electrons leads to a negative shift in V_th_. As shown in Equation (2), the Vo^0^(2H) states with two localized electrons are generated after Vo^0^ is substituted by H. The formed Vo^0^(2H) states can create filled defect gap states lying above the valence band edge. Therefore, the Vo^0^(2H) states could transition into Vo^2+^(2H) states with two free electrons under illumination excitation, leading to instability under NBTIS conditions [30]. Thus, the pronounced device instability and hydrogen sensitivity of BCE a-IGZO TFTs should be ascribed to the formation of a high density of substitutional H states, promoted by the high density of Vo^0^ defects resulting from back-channel damage.

Based on the above results, an improved BCE process was developed to enhance the stability of a-IGZO TFTs by removing the surface layer of the back channel, which contains a high density of Vo defects. In the conventional BCE process, the wet etching of S/D electrodes is usually conducted with F-free etchant to avoid over-etching of the a-IGZO film. In the improved process, the F-containing etchant was used for the wet etching of the S/D metal, and the around 25 nm thick surface layer of the back channel was removed. It is noted that the deposited thickness of the a-IGZO film in the improved process was set to be about 85 nm, instead of 60 nm in the conventional process, meaning that the final active layer thickness of the two kinds of TFTs is almost the same. In addition, the rest of the fabrication process of the above two TFTs were the same.

**Figure 4:**
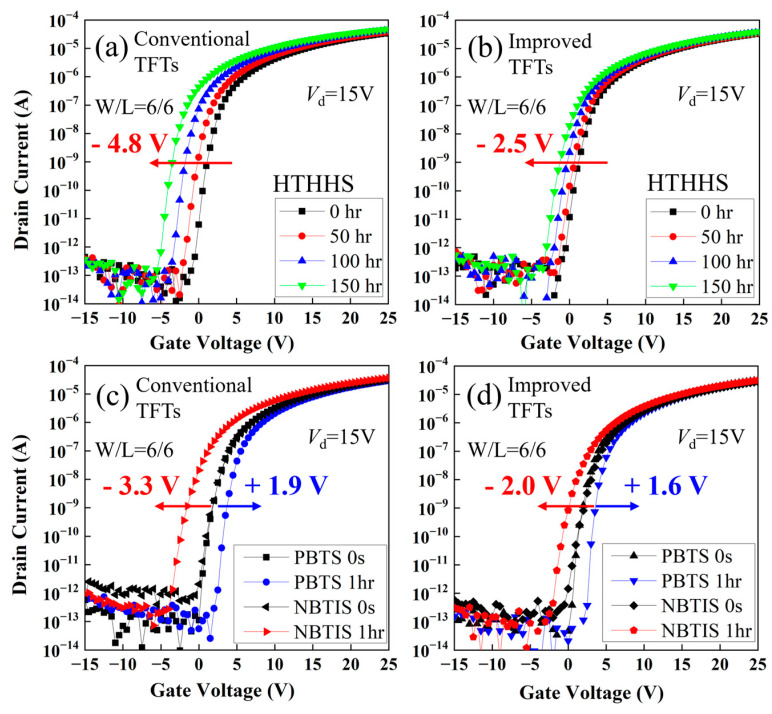
(**a**,**b**) Evolutions of transfer characteristics of the conventional and improved a-IGZO TFTs under HTHHS conditions. (**c**,**d**) Transfer characteristics of conventional and improved a-IGZO TFTs before and after PBTS/NBTIS stress.

As shown in Figure 4, the fabricated TFTs with the improved process exhibit a V_th_ shift of only −2.5 V after being under HTHHS at 60 °C and with 90% humidity for 150 h, and −2.0 V after being under NBTIS for 1 h. However, the conventional TFTs show much worse V_th_ shifts of −4.8 V and −3.3 V under the same HTHHS and NBTIS conditions, respectively. Thus, it is effectively demonstrated that removing the surface layer of the back channel could effectively alleviate the instabilities of BCE a-IGZO TFTs under HTHHS and NBTIS conditions. These results also testify that the high density of Vo defects at the back channel can significantly deteriorate the device stability of BCE a-IGZO TFTs.

**Figure 5:**
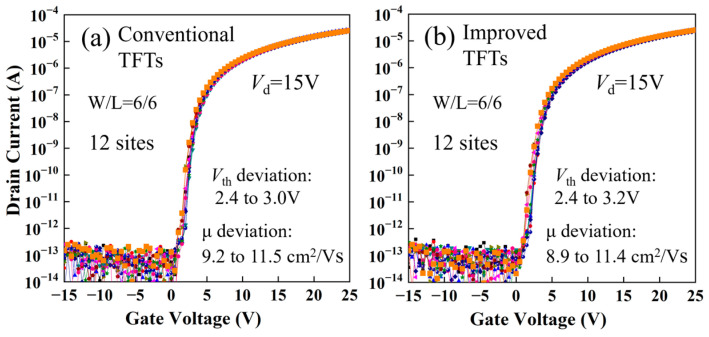
The transfer characteristics of the 12 fabricated TFTs on a G4.5 glass substrate, with (**a**) the conventional TFTs and (**b**) the improved TFTs.

To further compare the uniformity of the electrical characteristics of the conventional and improved TFTs, the transfer characteristics of the 12 fabricated TFTs on a G4.5 glass substrate were measured. As shown in Figure 5, for the improved TFTs, the V_th_ and mobility (μ) deviations are 0.8 V and 2.5 cm^2^/Vs, respectively, while for the conventional TFTs, they are 0.6 V and 2.3 cm^2^/Vs, respectively. The results indicate that removing the surface layer of the back channel by means of wet etching using F-containing copper etchant does not cause significant deterioration of the uniformity of electrical characteristics.

## 4. Conclusions

It is experimentally verified that the high density of Vo defects at the back channel of BCE a-IGZO TFTs result from the sputtered deposition rather than the wet etching process of S/D electrodes. Also, it is disclosed that the generated Vo defects are distributed within a depth range of about 25 nm on the back surface, and it is demonstrated that the surface layer containing a high density of Vo defects can be effectively removed by using F-containing copper etchant, leading to a remarkable improvement in device stability. As the etch method is quite convenient to implement in practical fabrication processes, it has already been applied in production lines recently. It is thus concluded that removing the surface layer containing the Vo defects is an effective solution to the device instability of BCE a-IGZO TFTs.

## Figures and Tables

**Table 1:** The sheet resistance (R_s_) of amorphous IGZO films after different processes.

Film	Process		R_s_	
	IGZO Annealing	S/D Sputtering	S/D Wet Etching	Log R_s_ (Ω/sq)
film-1	√	×	×	~9
film-2	√	×	F-free	~9
film-3	√	×	F-containing	~9
film-4	√	√	F-free	~4
film-5	√	√	F-containing	~7

## Data Availability

Data are contained within the article.
